# Solid-State Lithium-Ion Batteries as a Method for Doping Halide Perovskites with an *In Situ* Optical Readout of Dopant Concentration

**DOI:** 10.1021/jacsau.2c00212

**Published:** 2022-06-01

**Authors:** Angus Mathieson, Sascha Feldmann, Michael De Volder

**Affiliations:** † Institute for Manufacturing, Department of Engineering, University of Cambridge, 17 Charles Babbage Road, Cambridge CB3 0FS, United Kingdom; ‡ Cambridge Graphene Centre, Department of Engineering, University of Cambridge, 9 JJ Thomson Avenue, Cambridge CB3 0HE, United Kingdom; § Cavendish Laboratory, Department of Physics, University of Cambridge, 17 JJ Thomson Avenue, Cambridge CB3 0HE, United Kingdom

**Keywords:** perovskite, electrochemical, doping, LIB, Burstein−Moss, photoluminescence

## Abstract

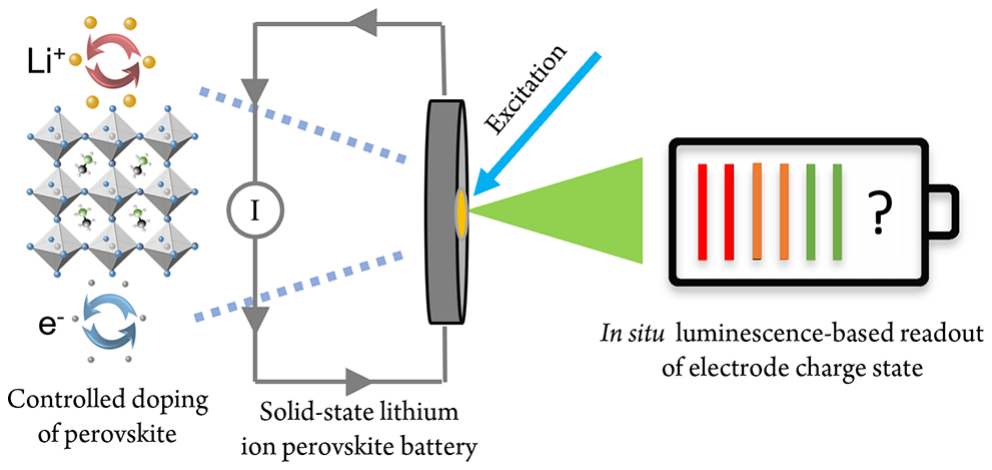

Controlled doping of halide perovskites is a longstanding challenge for efficient optoelectronic applications. Here, a solid-state lithium-ion battery (LIB) inspired device is used as a method of extrinsically doping a halide perovskite in a controlled and measurable fashion. The Burstein–Moss band gap shift induced by the electronic doping is measured using *in situ* optical spectroscopy to monitor the fraction of injected charges that successfully n-type dope the perovskite. By comparing the optical and electrochemical readouts of the charge density, we demonstrate a 96% doping efficiency during the insertion process. Subsequent charge removal steps demonstrate only a partial “undoping” of the perovskite, providing insights into the capacity degradation pathways in perovskite LIB electrodes.

Controlled charge or ion doping of semiconductors is a critical tool to enable the creation of devices with tailored optoelectronic properties.^1−4^ Halide perovskites (HPs) have gained attention as effective materials for solution-processable solar cells^5^ and light-emitting diodes (LEDs).^6^ They have demonstrated long charge carrier lifetimes,^7^ high optical absorption coefficients,^8^ and efficient light emission properties,^9,10^ which is surprising given the relative coarseness of their solution-based processing. However, controlled doping has hitherto proven difficult in this class of semiconductors.

Substitutional techniques, whereby the A site cation of the HP is replaced with Ag^+^, Rb^+^, or EA^+^ for example or the B site cation is replaced with Mn^2+^ or Cd^2+^, have demonstrated many viable approaches to tuning the properties of HPs.^11^ The addition of various metal ions, such as potassium^12^ and rubidium,^13^ has also shown different functional responses in perovskite thin films and crystals, including defect passivation, and a degree of control over the perovskite crystallization process during fabrication.

At present, cesium is the only widely employed alkaline metal to be actually incorporated into the crystal lattice.^14^ However, recent work has also shown how Li can be incorporated into the structure of CsPbBr_3_ in order to improve the charge injection efficiency in an LED,^15^ increase the photocurrent of a photovoltaic device, and even introduce diamagnetism in this material.^16^ Self-doping has been demonstrated in other HPs whereby nonstochiometric amounts of precursor compounds are added during the synthesis step, giving rise to excesses of specific species, such as iodide or bromide.^17^

Metal ions such as silver, strontium, and cerium have been shown to alter the electronic properties of the HP surface, changing the surface character from intrinsic to metallic by introducing n-type electronic states near the conduction band minimum when they are added to the precursor solution.^18^ However, large variations of local dopant concentration and structural position are commonly observed using this *in-synthesis* method. Ground-state molecular charge transfer techniques have been demonstrated as a means to n-type dope perovskite nanocrystals and quantum dots, due to their high surface area to volume ratio. However, this technique is not suitable for three-dimensional HP thin films and p- or n-type charge carrier doping that is both significant and well controlled has not yet been achieved in these systems.^19,20^

In this work we demonstrate the ability to dope a HP thin film using a simple lithium-ion-battery (LIB)-inspired device architecture, controllably inserting (and partially removing) Li ions and electrons into (and out of) the material. This is primarily useful as a single-stage doping process, whereby it is envisioned that a perovskite material may have an atomically precise number of Li ions and electrons added to the structure before being implemented into an end-use device. We also demonstrate how the dopants may be partially removed in subsequent cycles. The technique is based loosely on an electrochemical doping technique, employed in some organic semiconductor systems.^21^

Using *in situ* photoluminescence (PL) spectroscopy and applying the Burstein–Moss theory, we quantify the Li^+^ and e^–^ doping concentrations of the HP over multiple insertion and removal cycles and compare the values to those measured electrochemically. The comparison provides noninvasive insights into the state of charge of the battery, while also quantifying irreversible side reactions such as solid electrolyte interface (SEI) formation.^22^ From the multiplication of the applied current and time period, the number of Li^+^ and e^–^ species that move through the cell and are incident on the HP electrode may be calculated. By a comparison between this value and that calculated from the Burstein–Moss shift of the perovskite band gap—which represents the number of species that actually contribute to the doping of the perovskite—the number of dopants lost to noninsertion processes can be determined. This has significant implications extending beyond perovskite semiconductor devices and more broadly into battery science.

Galvanostatic charge/discharge processes were used to insert and remove Li ions and electrons (in a 1:1 ratio) from a HP electrode based on MAPbBr_3_ (MA = methylammonium) (Figure 1a). For this, a constant current of 0.150 ± 0.002 μA was applied for a predetermined length of time (Figure 1b). Note that a negative current indicates charge flow from the anode (Li metal) to the cathode (HP) in this half-cell battery configuration. During the lithiation process, Li ions are transported from the Li metal anode to the HP via the poly(ethylene oxide) (PEO) LiTFSI solid-state electrolyte while the electrons are injected through the external circuit.^23,24^ Previous attempts at producing a reliable HP LIB device have been hindered by the use of conventional liquid electrolytes comprising polar solvents that quickly dissolve the HP species. In this work the use of a solid-state polymer system removes all such complications and could encourage the use of this technique for related perovskite materials.

**Figure 1:**
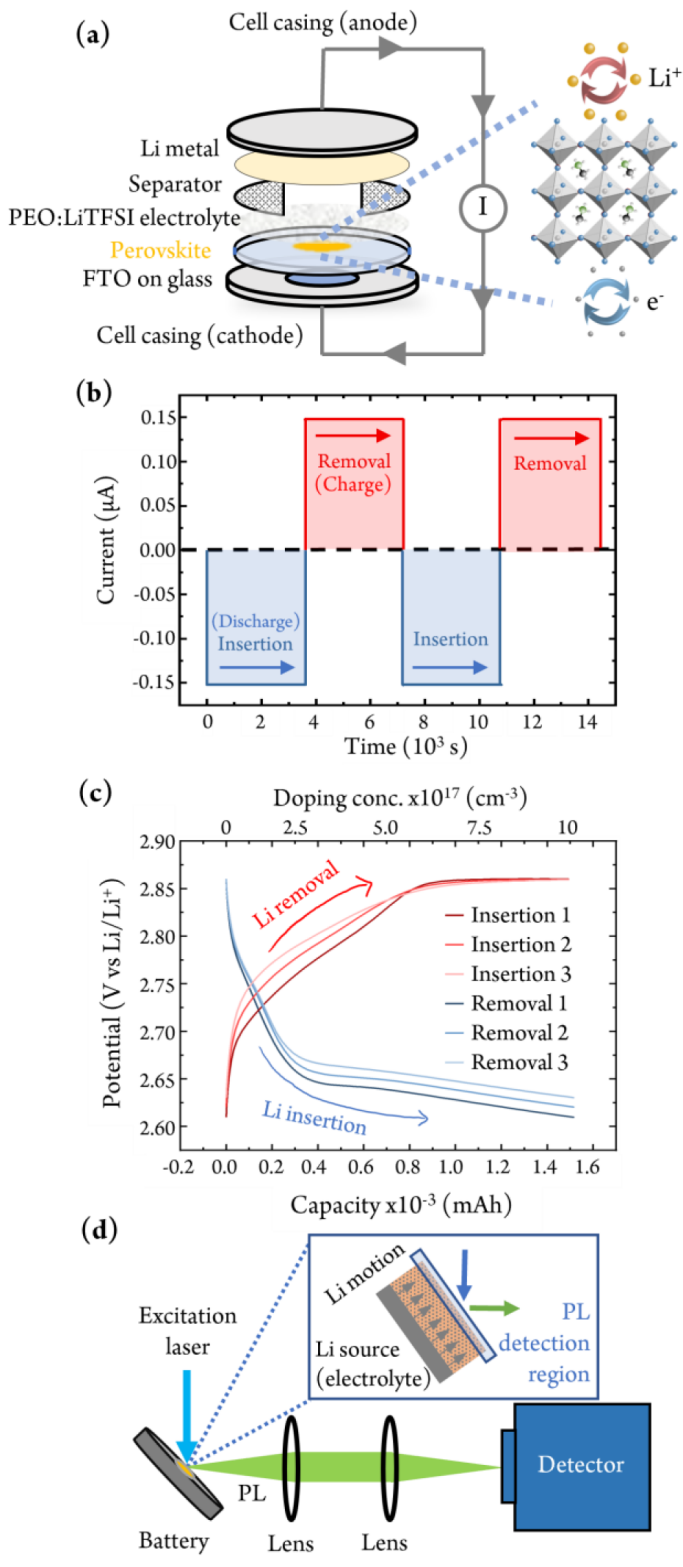
Controlled doping of a halide perovskite using a battery-inspired device architecture. (a) Schematic representation of the battery device stack used. Abbreviations: PEO, poly(ethylene oxide); LiTFSI, lithium bistrifluoromethanesulfonylimide; FTO, fluorine-doped tin oxide. (b) Applied current and cycling time during galvanostatic battery discharge (Li^+^ + e^–^ insertion) and charge (removal) cycles. (c) Galvanostatic charge–discharge curves of the LIB device showing three Li insertion and removal processes and the equivalent doping concentration after each step. (d) *In situ* photoluminescence (PL) spectroscopy setup to probe the battery at different charge doping states. Inset: schematic showing the PL detection region at the rear side of the perovskite relative to the Li insertion interface.

The doping mechanism involves electrons being injected directly into the conduction band of the HP, engendering the n-type doping of the material. Electrons and Li ions are generated by the anodic half-cell reaction
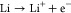
1while at the cathodic interface Li ions are simultaneously incorporated into the HP structure from the LiTFSI:PEO electrolyte, providing charge compensation. Note that the Li^+^ from LiTFSI → Li^+^ + TFSI^–^ is replenished by the Li^+^ generated in eq 1 such that all components, including the electrolyte, remain charge balanced.

The number of Li ions and therefore the electronic doping concentration were calculated from the knowledge that each coulomb of charge measured through the external circuit represents 6.24 × 10^18^ Li ions added to the HP. The applied current over time (Figure 1b) shows the multiple discharging (Li^+^ + e^–^ insertion into the HP) and charging (Li^+^ + e^–^ removal from the HP) processes (see also Figure S2 in the Supporting Information). The corresponding charge/discharge capacity curves are shown in Figure 1c. By keeping the potential above ∼2.0 V and within the first voltage plateau, we avoid the conversion and alloying electrochemical reactions according to previous publications.^24,25^ At each state of charge, the PL spectrum of the HP electrode was measured (see Figure 1d for the setup, Figure 2b for spectra, and Figure S6 for the digital image). To the best of our knowledge this represents the first time a PL measurement has been recorded on an operating battery electrode to quantify the number of ions that have been inserted. The PL emission is collected from the side of the perovskite farthest away from the Li source (the electrolyte and Li metal counter electrode). The spectra are therefore a result of Li diffusing throughout the perovskite and not just at the interface immediately in contact with the electrolyte (as shown in the inset in Figure 1d).

**Figure 2:**
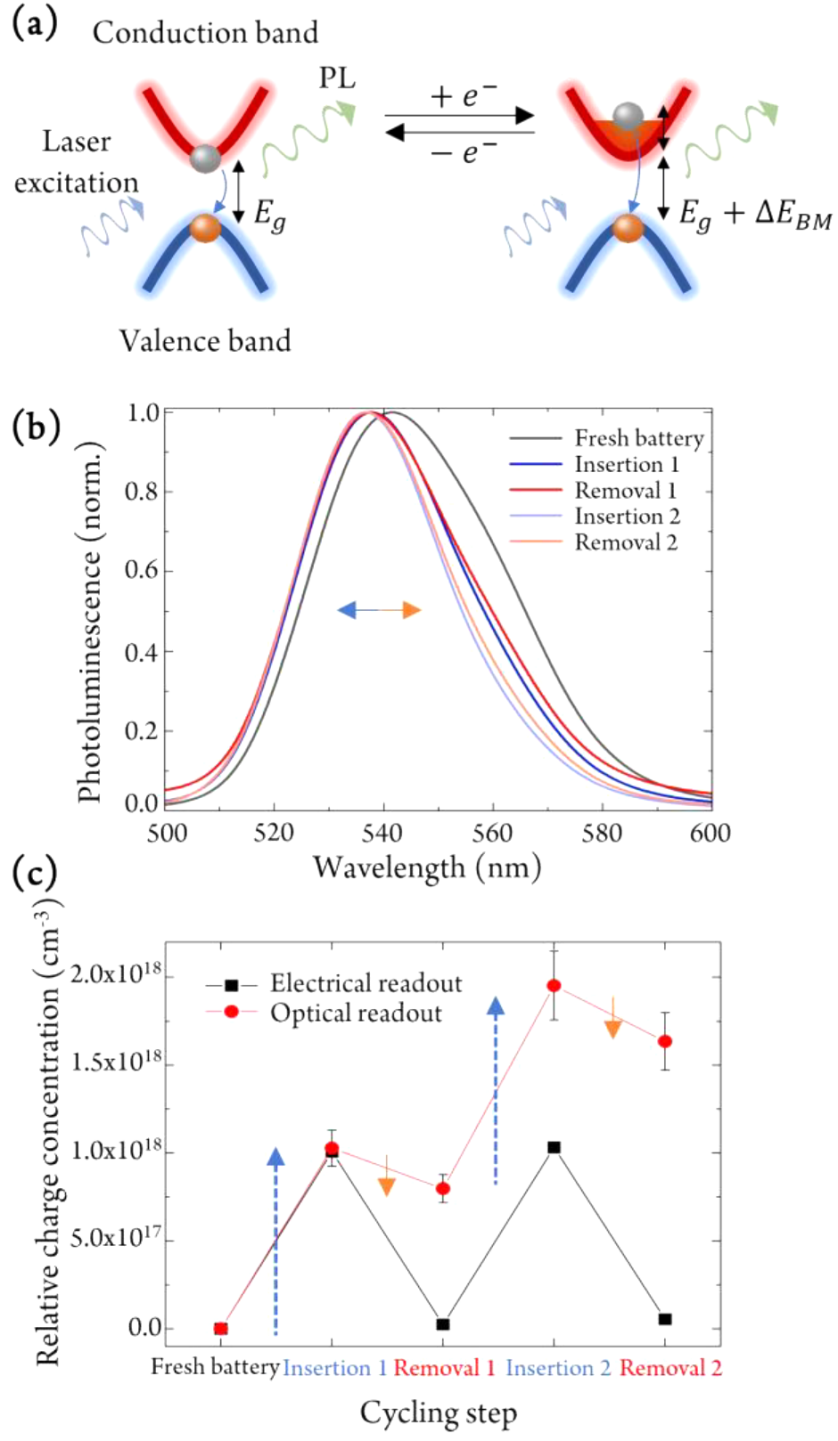
*In situ* determination of the HP doping concentration. (a) Sketch of Burstein–Moss (BM) induced PL changes, related to the charge density present in the battery at each stage of operation. Definitions: *E*_g_, band gap energy of the perovskite; Δ*E*_BM_, change in energy gap due to the BM effect. (b) *In situ* PL spectra of the battery at different cycling steps. (c) Doping concentration of the perovskite as extracted from PL data (red circles) and electrochemical readout (black squares) after each charge insertion and removal process. Arrows denote equal amounts of charge density added (blue) or removed (orange), revealing that losses mostly occur during the charge removal (i.e., recharging) step, while the n-type doping process is highly efficient.

We observe that the centroid of the PL spectrum blue-shifts to lower wavelengths (higher energy) during discharging (Li^+^ + e^–^ insertion) and reversibly red-shifts to higher wavelengths (lower energy) during charging (Li^+^ + e^–^ removal). This observation can be explained by the Burstein–Moss (BM) effect^26−28^ (Figure 2a), in which the injected electrons partially fill up available states in the conduction band. Photoexcitation of charge carriers from the valence into the conduction band results in the occupation of energetically higher lying states in comparison to the undoped case, and the recombination of conduction band electrons with valence band holes increasingly occurs from higher-energy states, resulting in a blue-shifted emission. Removal of the Li ions and electrons then reverses the process, restoring the original emission spectrum. The additional narrowing of the low-energy side of the PL spectrum upon doping can be explained by the filling of the lowest-energy states of the conduction band tail.^29^

The observed change in band gap (*E*_g_) due to the BM effect, Δ*E*_BM_, is related to the electron doping density (*n*_e_) approximately via
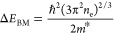
2where m* is the effective electron mass.^26,27^ This has been set to the literature value of 0.12 free electron masses for MAPbBr_3_.^30^ This relation is used to extract the doping density of added electrons and therefore Li ions at the different states of charge, which are then compared to the values extracted from the electrochemical measurements (Figure 2c). We note that the effective mass is the only parameter set in this fitting procedure.

We find that the optically measured doping density after the Li ion insertion processes calculated from the BM shift matches the value determined from electrochemical readout remarkably well and is reproducible (see Figure S2 in the Supporting Information for repeat measurements).

In addition to the PL spectral position changes, we also investigated the PL intensity and lifetime and observed that both of these decrease upon doping (see Figure S4 in the Supporting Information). For a highly ordered semiconductor, such as crystalline GaAs, one might intuitively expect the PL intensity to increase as a result of faster radiative recombination of minority holes with majority electrons,^31−34^ the concept of doping has still barely been explored in hybrid halide perovskites, as controlled charge doping has rarely been demonstrated. In this case, we expect a delicate interplay between trap density passivation and high recombination rates on the one hand combined with impurity-bound nonradiative and Auger recombination, as well as gradual degradation (see e.g. the material color change in Figure S5 in the Supporting Information) on the other to affect the overall PL intensity and lifetime charge.

Of the 3.36 × 10^15^ charges passing through the battery circuit, (3.2 ± 0.3) × 10^15^ (96%) were measured optically to have contributed to the doping of the perovskite (the corresponding charge densities are 1.0 × 10^18^ and 9.58 × 10^17^ cm^–3^, respectively). The subsequent removal of approximately the same amount of charge density (∼10^18^ cm^–3^) indicated by the potentiostat, however, does not match the lower concentration as determined by PL (∼3.2 × 10^17^ cm^–3^), suggesting an irreversibility during the removal (“undoping”) process. As the electrode also visibly degrades upon repeated cycling (Figure S5 in the Supporting Information), we conclude that the discrepancy in measured doping density is a result of oxidative side reactions during charge removal, for example the oxidation of halide or methylammonium ions, which could explain the increasingly darker color of the electrode. The PL- and electrochemistry-based charge density estimations for the second insertion process again match very well, indicating that the insertion process at this current density is highly efficient (compare the identical lengths of blue arrows in Figure 2c).

We conclude that the previously unexplored concept of using a battery-inspired approach is suitable for a single-step n-type doping process in perovskite materials, whereby precisely controlled amounts of metal and electronic doping may be achieved during the first discharge cycle. The subsequent incomplete charge removal cycle confirms previous results suggesting that there are processes native to halide perovskites that make them unsuitable for conventional LIB electrode materials.^25^ However, quantifying the irreversibility provides insights into these degradation mechanisms.

We therefore recommend electrochemical Li insertion as an elegant method to n-type dope perovskite materials in a manner that is of interest for both optoelectronic and energy storage applications. In addition, we demonstrate the use of PL spectroscopy as an accurate method of simultaneously measuring doping levels and the battery state of charge. In comparison to measuring only the electrochemical state of charge, the PL signal probes the concentration inserted into the bulk of the material directly, and therefore by deduction, how many charges are lost to side reactions. As such, we demonstrate a nondestructive *operando* method to elucidate the working and degradation mechanisms of photoactive LIB electrodes.

## Supporting Information Available

Methods and experimental details and additional data and discussions (PDF)
