# Supplementary material for: Solid-State Lithium-Ion Batteries as a Method for Doping Halide Perovskites with an In Situ Optical Readout of Dopant Concentration
Source: JACS Au. 2022 Jun 1;2(6):1313–7. doi: 10.1021/jacsau.2c00212 (PMC9241003; doi:10.1021/jacsau.2c00212)
Supplement: Supplementary file 1 — au2c00212_si_001.pdf [file au2c00212_si_001.pdf]

Supporting Information

Solid-State Lithium-Ion Batteries as a Method for Doping Halide Perovskites with an In Situ Optical Readout of Dopant Concentration

Angus Mathieson[1,2,3]*, Sascha Feldmann[3]*✉, Michael De Volder[1] ✉

[1] Institute for Manufacturing, Department of Engineering, University of Cambridge, 17 Charles Babbage Road, Cambridge CB3 0FS, United Kingdom

[2] Cambridge Graphene Centre, Department of Engineering, University of Cambridge, 9 JJ Thomson Avenue, Cambridge, CB3 0HE, United Kingdom

[3] Cavendish Laboratory, Department of Physics, University of Cambridge, 17 JJ Thomson Avenue, Cambridge, CB3 0HE, United Kingdom

✉ *sf561@cam.ac.uk; mfld2@cam.ac.uk*

** The authors contributed equally to the manuscript.*

Methods

<u>Perovskite material synthesis</u>

Powders of the $MAPbBr_3$ perovskite were prepared by using a slow evaporation technique. First, solutions of $MAPbBr_3$ were prepared by mixing stoichiometric amounts of the precursor powders MABr (>=99% purity, anhydrous, Sigma Aldrich™) and $PbBr_2$ (98% purity, Scientific Laboratory Supplies)) in dimethylformamide (DMF, 99% purity, anhydrous Sigma Aldrich™) under an argon atmosphere. The resulting solutions were stirred using a magnetic stirrer at 70°C for 1 hour to ensure total and homogeneous dissolution. After cooling, the solution was transferred to a vacuum oven and dried overnight at 60°C. The powder precipitate was collected and stored again under an argon atmosphere until further use.

<u>Perovskite electrode fabrication</u>

The $MAPbBr_3$ electrodes were deposited onto fluorine doped tin oxide (FTO) coated glass, cut to squares of side length ~1.2 cm. The perovskite powder described above was redissolved into DMF in a 1M concentration and then drop cast directly and then heated on a hot plate under an Argon atmosphere at 90°C for 24 hours. 6 μL of solution was drop cast, resulting in a final film thickness of approximately 28 μm.

Polyethylene oxide:LiTFSI polymer electrolyte fabrication

First, the PEO (PEO10: 600k molecular weight, Sigma Aldrich™) was dried under vacuum at 80°C and the LiTFSI salt under vacuum at 160°C. 1.25 g of PEO, 816 mg of LiTFSI and 11 mL of acetonitrile were mixed together under an argon atmosphere (<0.5 ppm H_2O, O_2) and stirred for 24 hours at 360 rpm, using a magnetic stirrer. The solution was degassed by pumping the solution in an open container, three times, using the small antechamber of the argon glovebox, in order to remove all of the bubbles in the solution. The solution was coated onto a sheet of Teflon™ using a doctor blade coater at room temperature, set to a thickness of 100 mill (2.54 mm). The film was dried for 48 hours at 70°C in the argon atmosphere using a hotplate.

Cell fabrication

A custom-built coin cell with an optical window was used to measure the *in situ* PL emission of the perovskite electrode under various stages of electrochemical doping. Coin cells (CR2032) were modified by drilling a hole of diameter d = 8 mm into the bottom casing.

The drop cast perovskite film was placed in the cell casing with the optical window (cathode side) and sealed into place using EPOXY (EVO-STIK). Thin strips of copper foil were used to contact the FTO surface to the coin cell casing. A glass fibre separator was cut into a hollow rectangular shape and placed such that the outer edge formed a window around the active perovskite area, yet provided a protective separation layer between the anode and cathode. A piece of the PEO film was cut to shape ~1.2 cm x 1.2 cm and placed over the entire assembly followed by a lithium metal chip, stainless steel spacer and spring. The anode coin cell casing was then placed on top and the entire assembly crimped together to a pressure of 1000 psi.

The carbon additives and binders used in conventional LIB cells are not used in this work, so as to ensure that the studied material remains as relevant for device applications as possible. Furthermore, we find that the conductivity and binding of the pure perovskite film to be inherently sufficient for doping purposes.

Electrochemical cycling

The as prepared cells were cycled using a BioLogic potentiostat in galvanostatic (constant current) mode. The cells were placed in a sealed oven and heated to 80°C for 30 minutes prior to any electrochemical doping process, to ensure the complete melting of the PEO electrolyte above its glass transition temperature ensuring suitable ionic conductivity. The cells were cycled at a low current density of 0.104 $\mu A\ cm^{-2}$, so as to allow the Li to diffuse evenly throughout the perovskite material.

Photoluminescence measurements

To measure the photoluminescence (PL) spectra of the perovskite at a given state of charge, the perovskite electrode was mounted in a customised optical coin cell, described above. PL spectra were recorded by an electrically gated intensified CCD camera (iCCD, Andor Star DH740 CCI-010) connected to a grating spectrometer (Andor SR303i). The pulsed output from a mode-locked Ti:Sapphire optical amplifier (Spectra-Physics Solstice, 1.55 eV photon energy, 80 fs pulse width, 1 kHz repetition rate) was used to produce 400 nm excitation *via* second harmonic generation in a β-barium borate crystal to photoexcite the perovskite battery through its optical window (see Supporting Figures S3 and S4). The slit width of the detector was set to 100 μm and no spectral filters were added to the emission beam path.

Time-resolved photoluminescence was measured using the same setup as described above, employing the electrical gating of iCCD camera using 2 ns gate stepping at 2 ns gate width.

At least one hour was left between the lithiation/delithiation processes and the PL measurement. This was done so as to ensure the total relaxation of the Li within the perovskite, allowing it to reach an equilibrated state of homogenous distribution.

Additional figures

Solid state PEO electrolyte characterisation

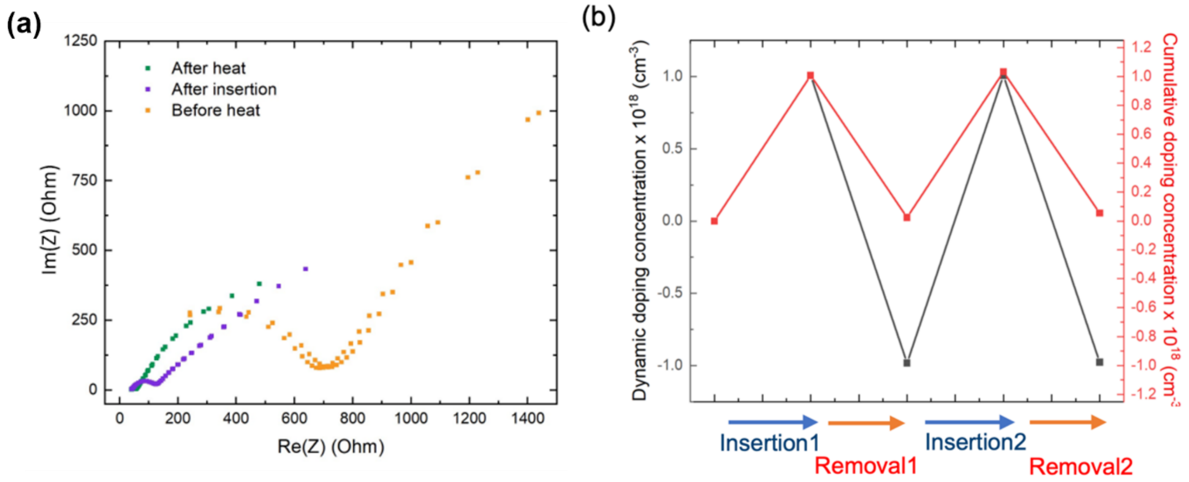

Supporting Figure S1: Extended electrochemical characterization of battery shown in main text. (a) Impedance spectroscopy of the $MAPbBr_3$ battery device before and after heating to the glass transition temperature of the PEO electrolyte (yellow – green) and after the first Li insertion cycle. (b) Comparison between the amount of Li^+/e^- inserted at each step (dynamic, left axis) and the cumulative amount (cumulative, right axis) following incomplete removal processes. (Ins = insertion of Li^+/e^-, Rem = removal of Li^+/e^- from the perovskites).

Fig. S1 (a) shows the electrochemical impedance spectroscopy characterizing the solid state perovskite battery device. The spectra show behaviour characteristic of the solid state polymer electrolyte before and after heating to the glass transition temperature. Immediately after assembling the cell, the charge transfer resistance is poor – due to a weak interface between the PEO and the perovskite. After heating, the charge transfer resistance drops significantly once the glass transition temperature (~70 °C) indicating the point above which the Li ions are mobilized. Furthermore, due to the melting of the PEO at this temperature, it is better able to impregnate the perovskite surface, resulting in an improved interfacial contact and therefore reduced charge transfer resistance.

Fig. S1 (b) compares the amount of Li inserted or removed during each step (dynamic doping concentration) with the net amount present in the material at any given time (cumulative doping concentration). Due to the fact that < 100 % of Li and therefore e^- are able to be removed from the electrode during each removal process, a discrepancy between the cumulative and dynamic doping concentrations is observed. From a battery device point of view, this corresponds to a Coulombic efficiency of < 100 %.

Additional cycling – pushing the material beyond the sensible doping concentration limit

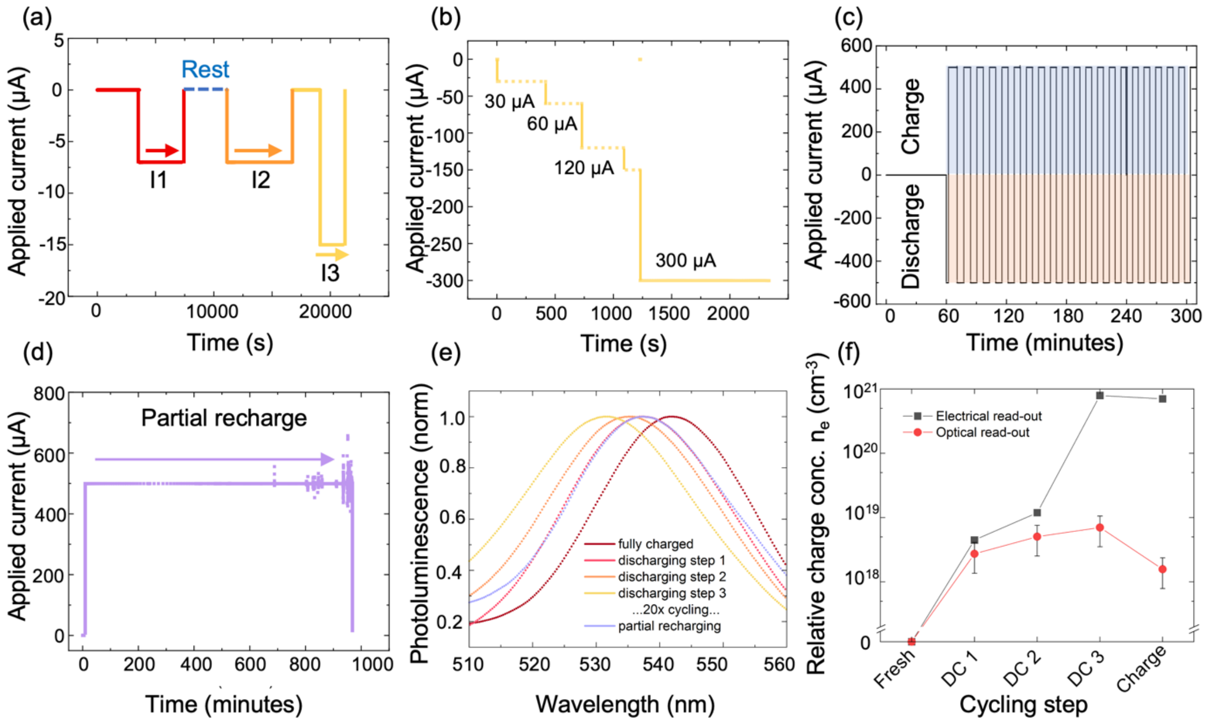

Supporting Figure S2: Additional data set of another battery sample with different charging program, involving much higher charge densities to test the limit of the process before breakdown. (a) First three insertion (discharge) processes. (b) Continuation of the third discharge process. (c) 20 charge and discharge cycles to test the long term cycling effects on the battery system. (d) Partial recharging of the cell to test the recovery of the red shift after long term cycling. (e) PL spectra taken after each of the steps mentioned above. (f) Comparison between the doping concentration as determined electrochemically vs from the Burstein-Moss model of the PL emission for the cell with the more aggressive cycling regime. (DC = discharge, Charge here = partial recharging after 20 cycles).The increasing divergence at higher charge densities and repeated cycling suggests successive degradation of the battery.

Fig. S2 summarizes additional measurements carried out, in which the response of the perovskite material, when exposed to extreme levels of doping and up to 20 repeated insertion/removal cycles is tested. Fig. S2 (a-d) describe the applied current conditions in chronological order of this experiment. First, -7.5 µA is applied for 2.5 hours in order to induce a dopant concentration of 5 x 10^{18} cm^{-3} (already 5x times higher than the concentrations used in the main text). This process is labelled I1 and is shown in red. Second, the cell was allowed to rest, in order to give time for the inserted Li to reach a state of spatial equilibrium within the perovskite and the PL spectrum measured. This process was then repeated and is labelled I2. Next, a large current starting at -15 µA and being increased stepwise to -300 µA (Fig. S2 (b)) was applied in order to insert dopants to a final concentration of 1 x 10^{21} cm^{-3}. Following this, the cell underwent multiple (20x) insertion and removal cycles in quick succession using a discharge/charge current of ±500 µA respectively in order to test the effect on the perovskite. This stage concluded the “insertion” investigation and was followed by a prolonged “removal” stage whereby

a charge current of 500 μA was applied – in order to test how much of the previously inserted species could be removed.

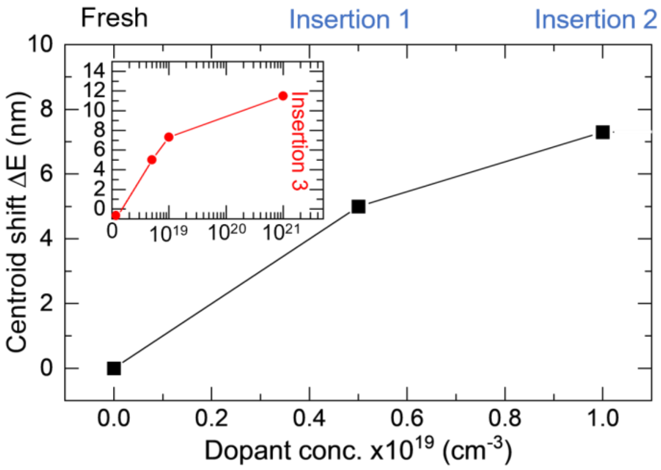

Supporting Figure S3: PL emission spectral shift as a function of inserted dopant concentration. First two processes shown in main panel with third process to a dopant concentration of 10^{21} cm^{-3} shown inset on logarithmic axis.

The effect of each of these processes on the PL spectrum of the perovskite are shown in Fig. S2 (e). Using the spectra of S2 (e), the dopant concentration determined by measuring the Burstein Moss shift is calculated and compared to the known number of dopants inserted electrochemically – the results of which are shown in Fig. S2 (f). As before, the first insertion process engenders a strong agreement between the observed dopant concentration and the number inserted, however, as before a disparity opens up in subsequent cycles. The magnitude of the discrepancy is seen to increase with a greater level of dopant concentration. When the material is pushed to an extreme dopant concentration of 10^{21} cm^{-3} there is little correlation between the observation based on the Burstein Moss shift and the intercalated dopants, indicating that processes in addition to simple injection of electronic charges take place.

Fig. S3 shows the shift of the PL emission as a function of inserted dopant concentration for the cell that is pushed to an extreme degree. The equivalent shifts recorded for the cell referred to in the main text can be found in Fig. S4 below.

Additional (time resolved) PL characterization

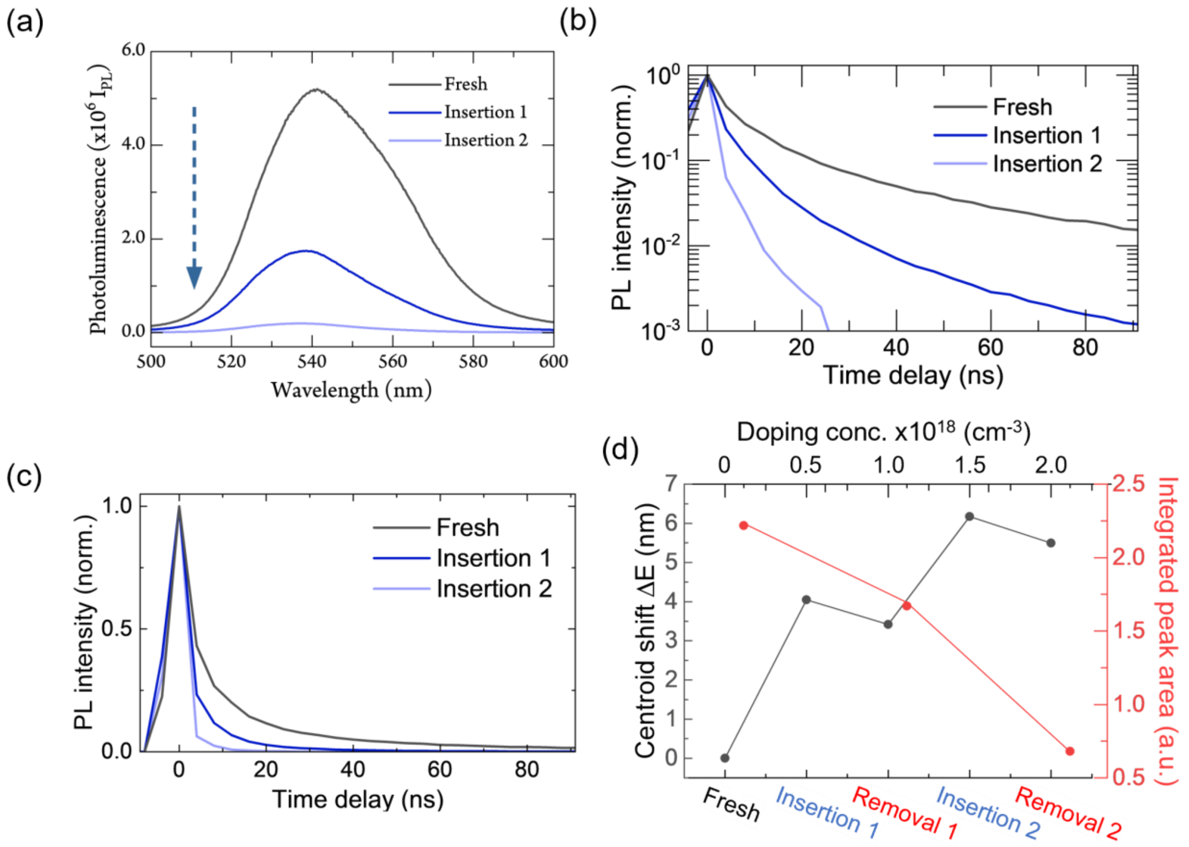

Supporting Figure S4: Intensity- and time-resolved PL measurements at different states of doping for cell 1. (a) Non-normalized PL emission spectra at various dopant concentrations. (b) Time resolved PL at different states of doping. (c) Time resolved PL (normalized). (d) Effect of dopant concentration on the PL emission peak shift and integrated intensity after the insertion/removal processes described in the main text. Excitation wavelength is 400 nm and the kinetics were integrated over the full PL spectrum.

Fig. S4 shows additional PL characterization of the cell discussed in the main text. With increasing doping concentration, the PL intensity and lifetime decreases, as most photo-excited holes become rapidly quenched by majority electrons. The drop in intensity is shown clearly in Fig. S4 (a) and the decreasing charge carrier lifetimes are shown in Fig S4 (b, c). Fig. S4 (d) shows how the two main parameters, the PL emission shift and integrated intensity vary with dopant concentration. The wavelength of the emission is observed to shift by up to 7 nm, as described in the main text. We note that this shift is similar to the cell that was pushed to extreme dopant concentrations, at least for the ranges that overlap (up to ~10^{18} cm^{-3}).

Visible color change of perovskite after excessive doping

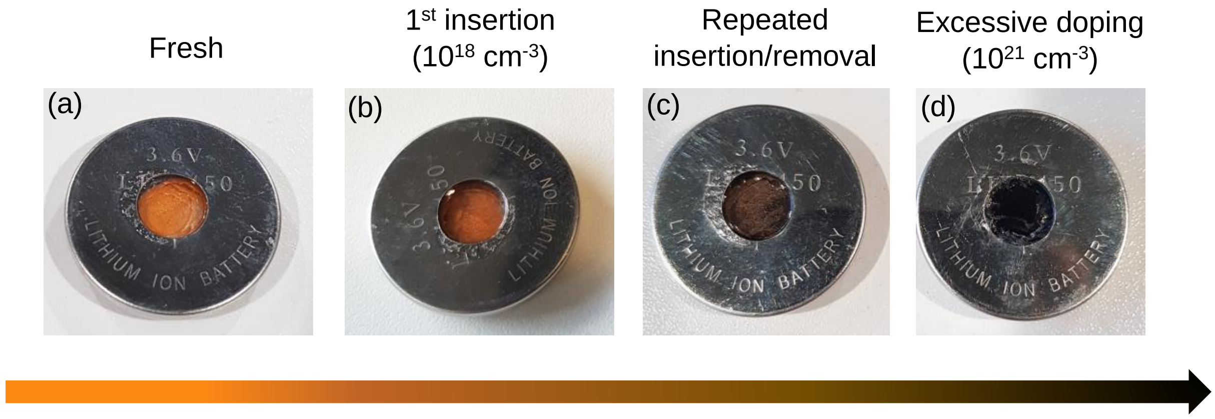

Supporting Figure S5: Photographs of battery with exposed active perovskite material, at various stages of cycling. (a) Fresh $MAPbBr_3$ cell as assembled, before any electrochemical processing. (b) Cell after the first insertion process to a dopant concentration of 1.0 x 10^{18} cm^{-3}. (c) Cell after the multiple insertion/removal processes described in the main text. (d) Cell pushed past the sensible doping regime. The final OCP of the cell shown was 0.1 V vs Li/Li^+. Black coloring is attributed to the formation of species from redox side reactions, including metallic lead, which has a known reduction reaction below 1.5 V vs Li/Li^+.

Fig. S5 shows digital photographs of the perovskite cell at various stages of Li insertion. Initially the perovskite in the cell exhibits the bright orange color characteristic of $MAPbBr_3$, as shown in (a). After the first insertion process, resulting in a dopant concentration of 10^{18} cm^{-3}, the same orange color is observed (Fig. (b)). However, although only subtle, the bright orange appears slightly dull after this step. Fig. (c) shows the appearance of the cell after the multiple insertion/removal steps and the perovskite now exhibits a considerably more dull orange/brown color. Finally, for the purpose of completion, after the cycling protocol described in the main text, a current/time protocol is applied such that dopants amounting to a concentration of 10^{21} cm^{-3} are inserted. Following this process, the cell exhibits the black color as shown in Fig. (d). We attribute this, given that the process required involved undergoing various electrochemical processes – demonstrated by flat plateaus in the galvanostatic discharge curves – to the reduction of the Pb^{2+} cation to metallic lead. However, we emphasize here that in order to achieve this, the cell must be pushed past any sensible level of discharge.

Photograph of cell during PL characterization

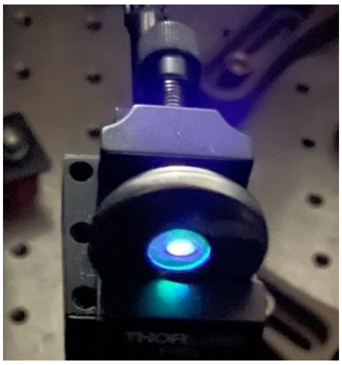

Supporting Figure S6: Photograph of $MAPbBr_3$ perovskite lithium-ion battery showing characteristic green photoluminescence upon photoexcitation at 400 nm.
